# Supplementary figures and images for: Melatonin increases growth properties in human dermal papilla spheroids by activating AKT/GSK3β/β-Catenin signaling pathway
Source: PeerJ. 2022 May 18;10:e13461. doi: 10.7717/peerj.13461 (PMC9123888; doi:10.7717/peerj.13461)

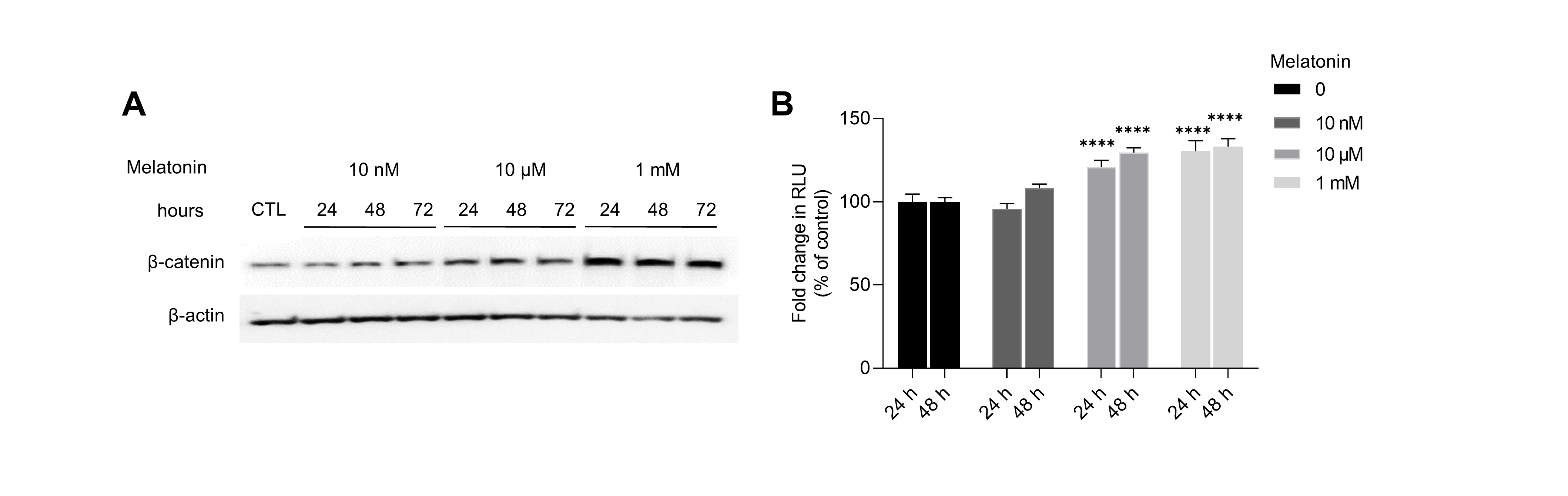

Supplement: Supplemental Information 1 — (A) HDP cells were treated with 10 nM, 10 µM, and 1 mM melatonin for 24 h, 48 h, and 72 h. β-catenin stabilization was assessed by western blotting and β-actin served as a loading control. (B) 293T cells were treated with 10 nM, 10 µM, and 1 mM melatonin for 24 h, 48 h. TCF/LEF transcriptional activity was determined using a luciferase assay and normalizing luciferase activity to β-galactosidase activity. Data are presented as the mean ± SD of three independent experiments. Two-way ANOVA, followed by Turkey’s post hoc test; **** p < 0.0001 versus DMSO treated control. [file peerj-10-13461-s001.png]

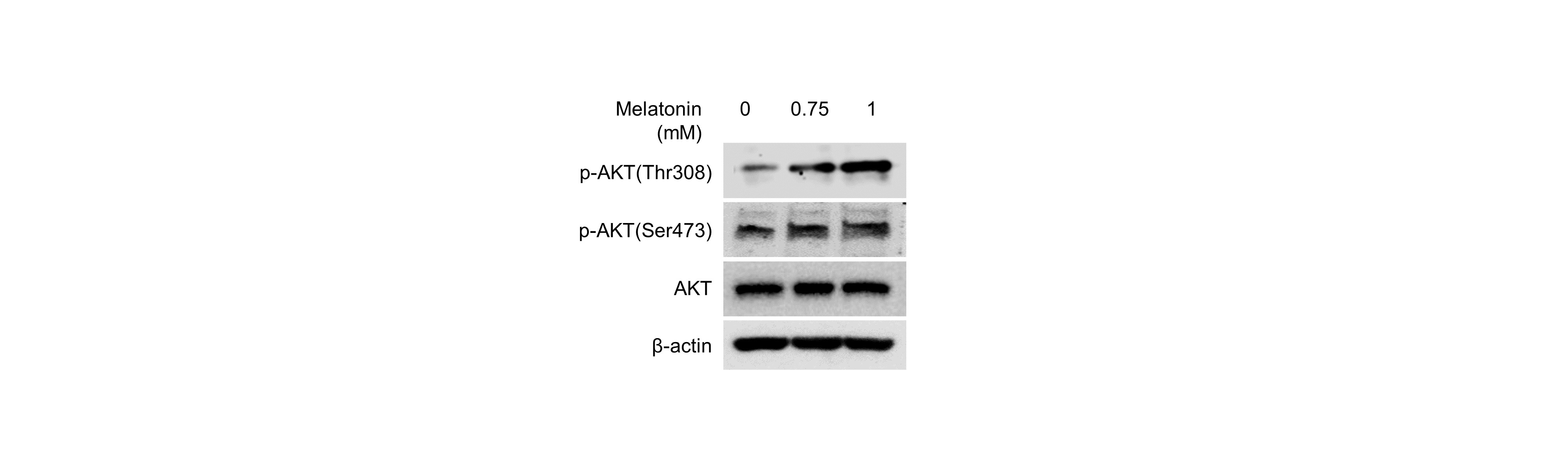

Supplement: Supplemental Information 2 — HDP cells were treated with 0.75 and 1 mM melatonin for 24 h. Protein levels of AKT phosphorylation were assessed by western blotting. β-actin served as a loading control. [file peerj-10-13461-s002.png]

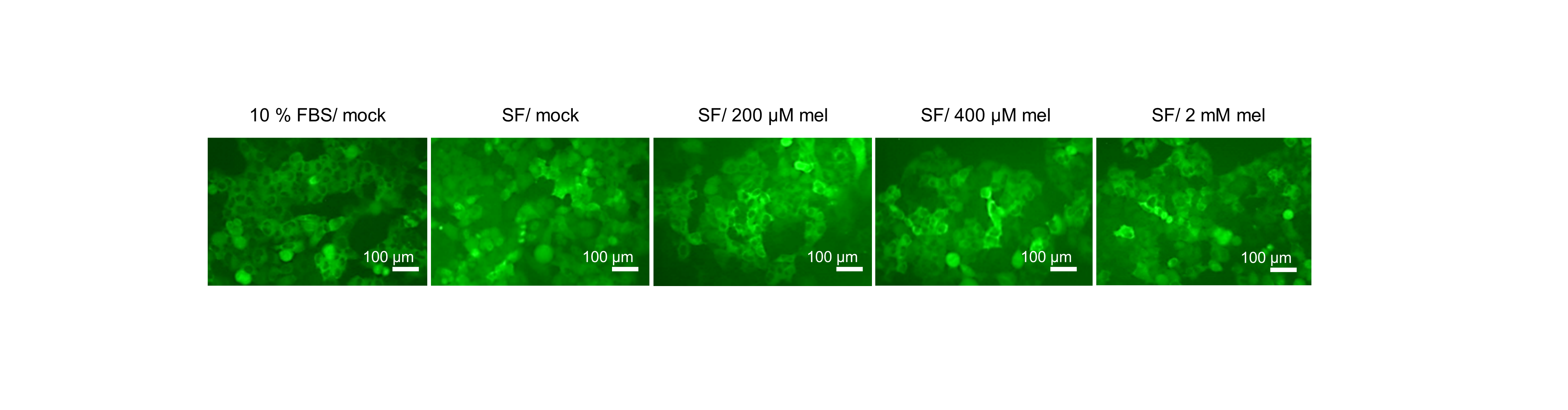

Supplement: Supplemental Information 3 — HeLa/FoxO1-Clover cells, capable of visualizing the AKT activity by FoxO1-tagged Clover fluorescent reporter, were treated with melatonin (0, 200, 400 µM, and 2 mM) under serum starvation conditions for AKT inhibition for 24 h. The subcellular localization of FoxO1-Clover was observed using an UV microscope Axiovert 200. The scale bars represent: 100 µm. [file peerj-10-13461-s003.png]
