# Supplementary material for: Melatonin increases growth properties in human dermal papilla spheroids by activating AKT/GSK3β/β-Catenin signaling pathway
Source: PeerJ. 2022 May 18;10:e13461. doi: 10.7717/peerj.13461 (PMC9123888; doi:10.7717/peerj.13461)
Supplement: Supplemental Information 6 [file peerj-10-13461-s006.pdf]

Supplement Materials & Methods

Cell Culture and Reagents

pLenti-FoxO1-Clover was received as a gift from Peter Rotwein (Addgene plasmid # 67759) (Gross & Rotwein 2015), pCMV delta R8.2 was a gift from Didier Trono (Addgene plasmid # 12263), and pVpack-VSV-G was purchased from Stratagene (La Jolla, CA, USA).

Cell Fractionation Analysis

Total cell lysates were prepared and protein extracts were obtained after lysis in RIPA buffer (25 mM Tris-Cl, 5 mM ethylenediaminetetraacetic acid, 150 mM NaCl, 1% NP40, 1% sodium deoxycholate, 0.025% sodium dodecyl sulfate) containing 200 mM phenylmethylsulfonyl fluoride.

Luciferase Reporter Assay

293T cells (1×10^5 cells/well) were seeded and cultured for 24 h. The cells were then co-transfected with the TCF/LEF promoter-driven luciferase reporter plasmid and pSV-β-galactosidase (pSV-β-gal) plasmid using Lipofectamine reagent (Thermo Fisher Scientific). Four hours post-transfection, cells were treated with melatonin (10 nM, 10 μM, and 1 mM). At 24 and 48 h post-treatment, transfected cells were lysed using passive lysis buffer (Promega). To determine luminescence, the cell lysates were incubated with d-luciferin (Sigma-Aldrich) and luciferase activity was measured using a Synergy HTX Multi-Mode Microplate Reader (Bioteck). Beta-galactosidase activity was measured using the Luminescent β-galactosidase detection kit II (Takara Bio Inc., Tokyo, Japan). Relative luciferase activity was calculated by normalizing luciferase activity to β-galactosidase activity.

Generation of AKT-Reporter Stable HeLa Cell Lines

The HeLa/FoxO1-Clover cell line capable of visualizing AKT activity by a FoxO1-tagged Clover fluorescent reporter was established by lentiviral transduction. Briefly, 293T cells (5×10^5) were transfected with pLenti-FoxO1-Clover (Addgene plasmid # 67759), pVpack-VSV-G (Stratagene), and pCMV delta R8.2 (Addgene plasmid #12263) plasmids using Lipofectamine reagent (Thermo Fisher Scientific, Waltham, MA, USA), and viral supernatants were harvested after 48 h. HeLa cells (2×10^5) were subsequently transduced and selected using 1 μg/mL puromycin (Amresco, Solon, OH, USA) for 2 weeks. For the experiments, HeLa/foxO1-Clover cells were seeded (1×10^5) in 24 well plates. Cells were then treated with melatonin (0, 200, 400 μM, and 2 mM) under serum starvation conditions for AKT inhibition. After 24 h, the subcellular localization of Foxo1-Clover was observed using an ultraviolet (UV) microscope Axiovert 200 (ZEISS, Germany).

References

Gross SM, and Rotwein P. 2015. Akt signaling dynamics in individual cells. *Journal of cell science* 128:2509-2519.
